# Supplementary material for: Role of cyclooxygenase-2-mediated prostaglandin E2-prostaglandin E receptor 4 signaling in cardiac reprogramming
Source: Nat Commun. 2019 Feb 20;10:674. doi: 10.1038/s41467-019-08626-y (PMC6382796; doi:10.1038/s41467-019-08626-y)
Supplement: Supplementary file 1 — Supplementary Information [file 41467_2019_8626_MOESM1_ESM.pdf]

SUPPLEMENTARY INFORMATION

Role of Cyclooxygenase-2/Prostaglandin E2/Prostaglandin E Receptor 4 Signaling in Cardiac Reprogramming.

Muraoka et al.,

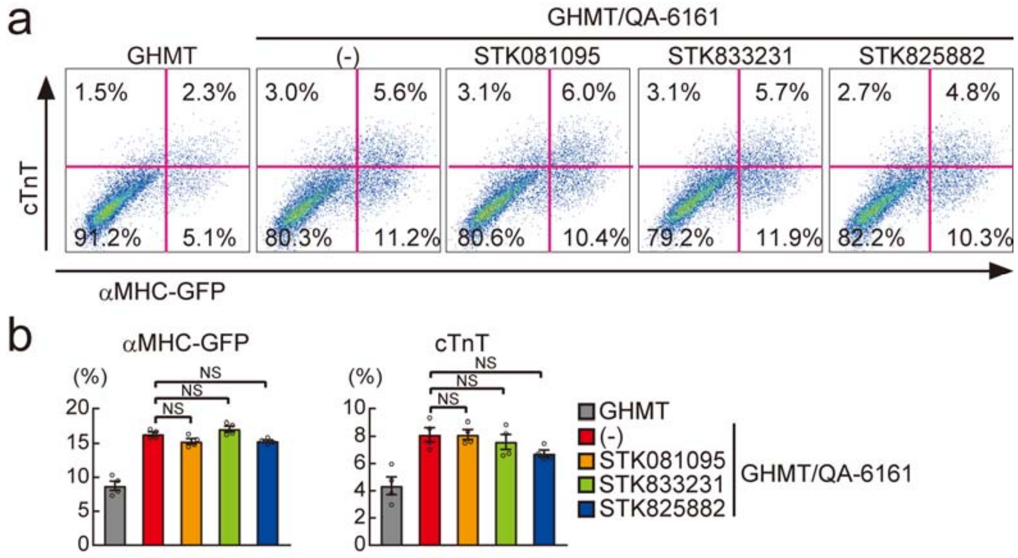

Supplementary Figure 1. Related to Fig. 1d.

Addition of Other Compounds Did Not Improve Diclofenac-induced Cardiac Reprogramming.

(a, b) FACS analyses for αMHC-GFP and cTnT expression in GHMT/QA-6161-treated TTFs with or without indicated compounds for 1 week. Quantitative data are shown in (**b**); n = 4 biologically independent experiments.

All data are presented as mean ± SEM. NS, not significant.

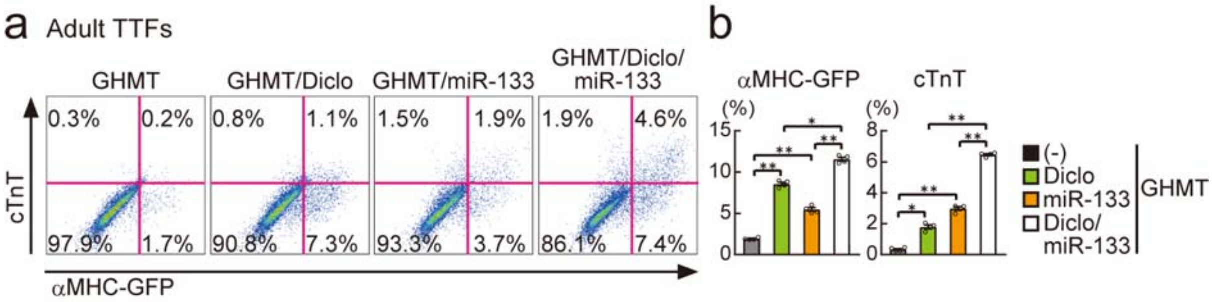

Supplementary Figure 2. Related to Fig. 3a.

Addition of miR-133 Promoted Diclofenac-induced Cardiac Reprogramming in Adult TTFs.

(a, b) FACS analyses for αMHC-GFP and cTnT expression in GHMT-transduced adult TTFs treated with diclofenac, miR-133 or diclofenac plus miR-133 for 1 week. Quantitative data are shown in (**b**); n = 4 biologically independent experiments.

All data are presented as mean ± SEM. *, $P < 0.05$, **, $P < 0.01$ vs. the relevant control (one-way ANOVA).

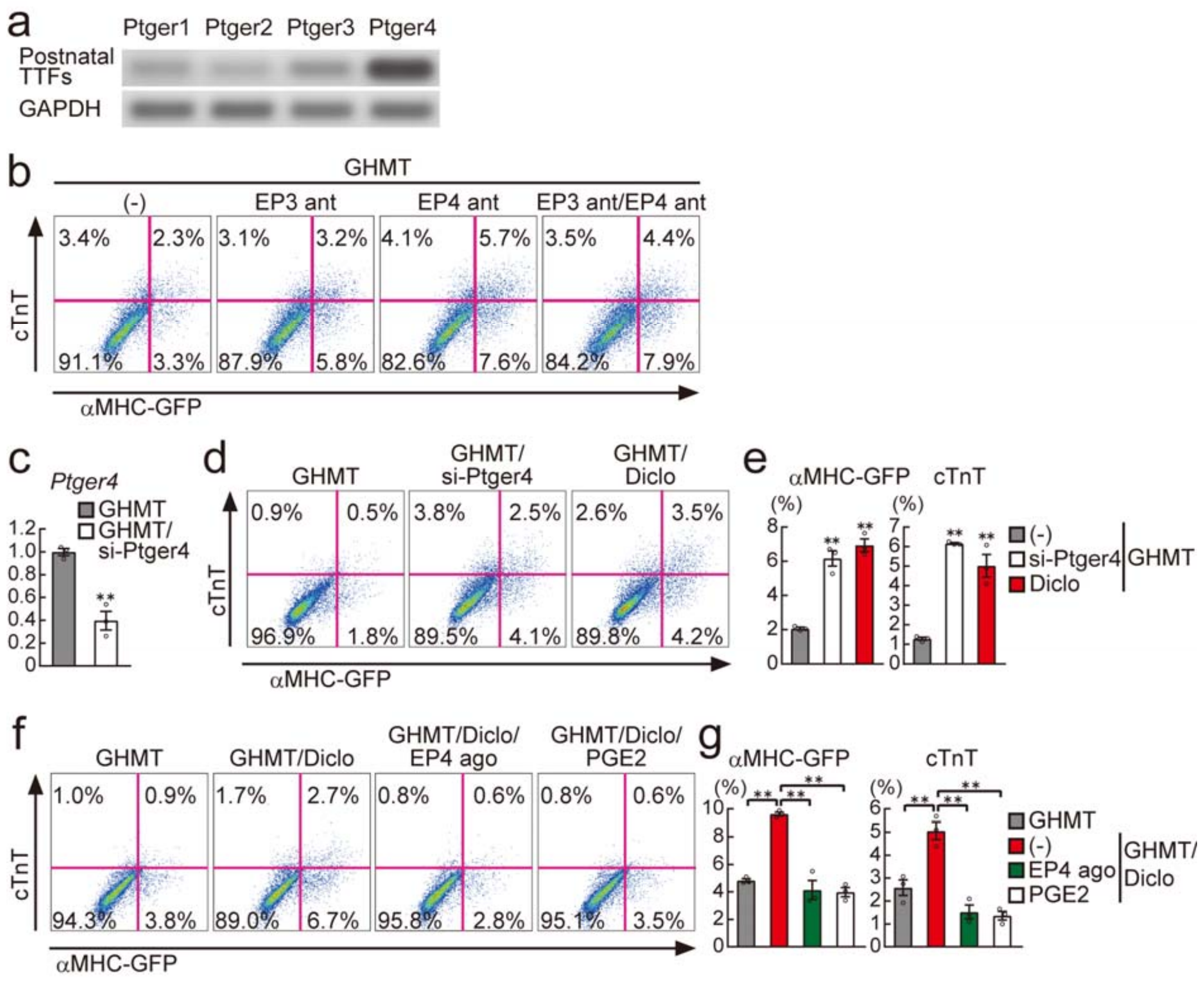

Supplementary Figure 3. Related to Fig. 4.

Inhibition of PGE Receptor 4 Improved Cardiac Reprogramming

(a) Semiquantitative RT-PCR for *Ptger1-4* in postnatal TTFs. Source data are prvided in **Supplementary Figure 7.**

(b) FACS analyses for αMHC-GFP and cTnT expression. GHMT-transduced postnatal TTFs were cultured with the indicated EP antagonists (ant) for 1 week.

(c) Relative mRNA expression of *Ptger4* in GHMT-TTFs transfected with scramble siRNA or siRNA against *Ptger4* (si-Ptger4); n = 3 biologically independent experiments. **, $P < 0.01$ vs. the relevant control (Student's *t* test).

(d, e) FACS analyses of αMHC-GFP$^+$ and cTnT$^+$ cells in postnatal TTFs 1 week after GHMT transduction with or without si-Ptger4. Quantitative data are shown in (**e**); n = 3 biologically independent experiments. **, $P < 0.01$ vs. the relevant control (one-way ANOVA).

(f, g) FACS analyses of αMHC-GFP$^+$ and cTnT$^+$ cells. GHMT-transduced TTFs were cultured with diclofenac with or without EP4 agonist (ago) or PGE2 for 1 week. Quantitative data are shown in (**g**); n = 3 biologically independent experiments. **, $P < 0.01$ vs. the relevant control (one-way ANOVA). All data are presented as mean ± SEM. NS, not significant. Diclo, diclofenac.

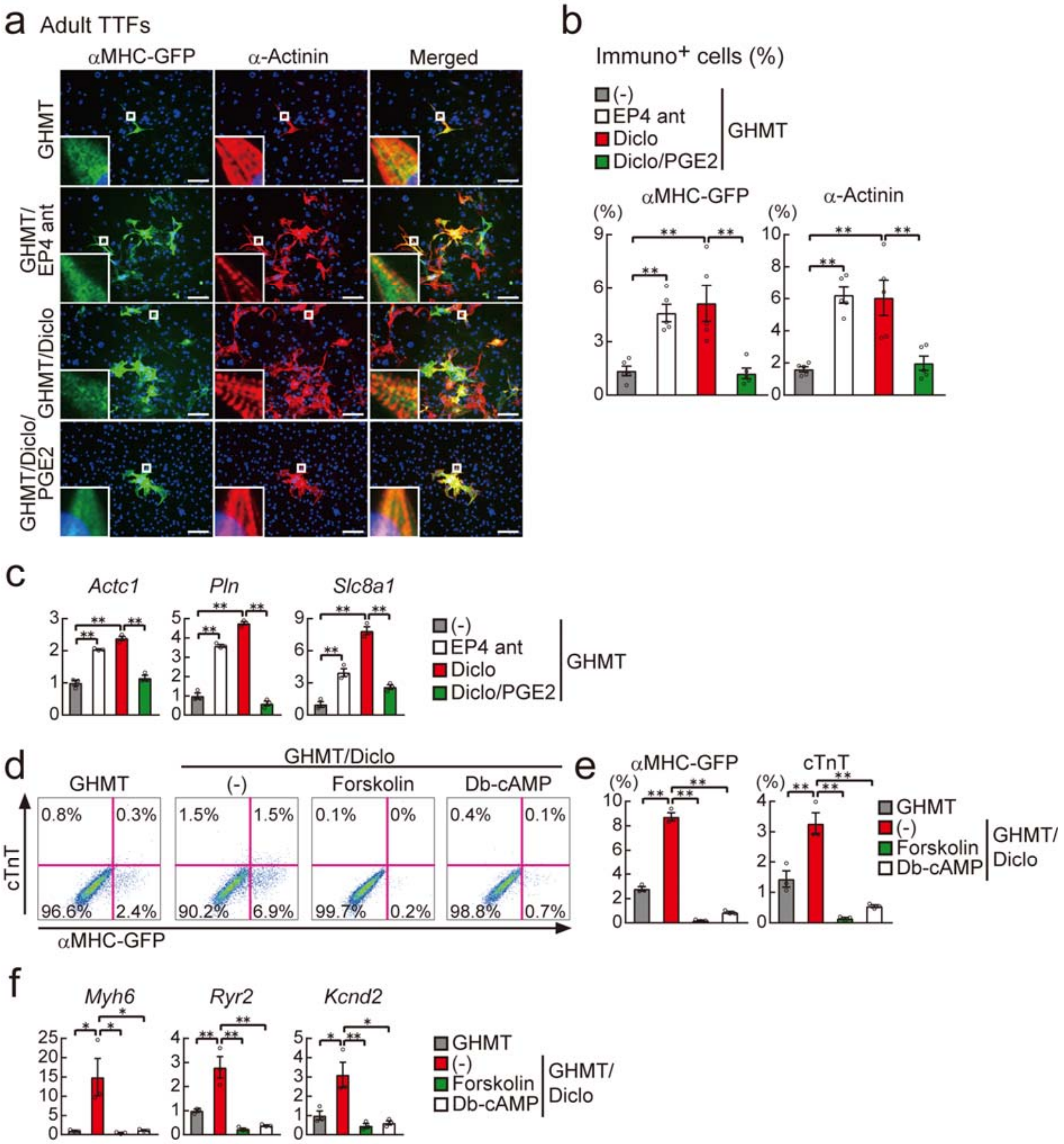

Supplementary Figure 4. Related to Fig. 5a.

EP4 Antagoinst Promoted Cardiac Reprogramming in Adult TTFs.

(a, b) Immunocytochemistry for αMHC-GFP, α-actinin, and DAPI. EP4 antagonist (ant) and diclofenac treatment increased generation of iCMs, while PGE2 inhibited GHMT/diclofenac-induced cardiac reprogramming in adult TTFs after 4 weeks. High-magnification views (insets) show the sarcomeric organization. Quantitative data are shown in (**b**); n = 5 biologically independent experiments.

(c) qRT-PCR analyses for cardiac gene expression in postnatal TTFs transduced with GHMT and

treated with or without indicated reagents for 1 week; n = 3 biologically independent experiments. **(d, e)** FACS analyses for αMHC-GFP$^+$ and cTnT$^+$ cells induced from adult TTFs. GHMT-transduced adult TTFs were cultured with diclofenac with or without forskolin or dibutyryl-cAMP (Db-cAMP) for 1 week. Quantitative data are shown in (**e**); n = 3 biologically independent experiments. **(f)** qRT-PCR analyses of cardiac gene expression in adult TTFs treated as indicated for 1 week; n = 3 biologically independent experiments. All data are presented as mean ± SEM. *, $P < 0.05$, **, $P < 0.01$ vs. the relevant control (one-way ANOVA). Scale bars represent 100 μm.

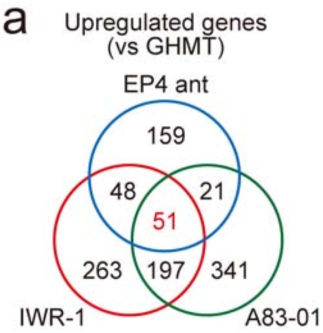

b GO categories enriched in GHMT/EP4 ant, GHMT/A83-01 and GHMT/IWR-1 Upregulated genes (51)

GO term	Genes	Size	p-value
myosin complex	7	61	1.3E-8
actin cytoskeleton	9	205	5.7E-8
myofibril	6	91	4.3E-6
contractile fiber	6	95	5.3E-6
myosin filament	4	15	8.2E-6
muscle system process	5	67	1.5E-5
motor activity	6	138	2.8E-5
sarcomere	5	80	6.2E-5
contractile fiber part	5	83	8.3E-5
blood circulation	5	111	1.1E-4

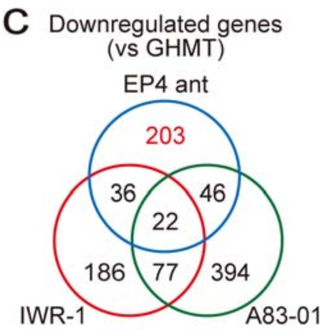

d GO categories enriched in GHMT/EP4 ant specifically downregulated genes (203)

GO term	Genes	Size	p-value
extracellular region	40	1680	3.9E-6
defense response	18	448	6.9E-6
extracellular region part	24	774	1.4E-5
response to peptide hormone stimulus	7	95	5.7E-4
proteinaceous extracellular matrix	11	297	1.8E-3
immune response	14	471	1.8E-3
3',5'-cyclic-nucleotide PDE activity	4	24	2.3E-3
extracellular matrix	11	309	2.4E-3
cyclic-nucleotide PDE activity	4	25	2.6E-3
inflammatory response	9	225	3.1E-3

Supplementary Figure 5. Related to Fig. 6h.

EP4 Antagoinst Promoted Cardiac Reprogramming via Suppression of Inflammation and Fibroblast Signatures.

(a, b) Venn diagram showing the genes that are upregulated in postnatal TTFs with EP4 antagonist (ant, blue), GHMT/TGFβ inhibitor (A83-01, green) or GHMT/Wnt inhibitor (IWR-1, red) by more than 2-fold compared to GHMT alone, after 1 week. Top 10 GO analyses for the 51 upregulated genes with EP4 antagonist, TGF-β inhibitor, and Wnt inhibitor treatment are shown in (**b**).

(c, d) Venn diagram showing the genes that are downregulated in postnatal TTFs with EP4 antagonist (ant, blue), GHMT/TGF-β inhibitor (A83-01, green) or GHMT/Wnt inhibitor (IWR-1, red) by more than 2-fold compared to GHMT alone, after 1 week. Top 10 GO analyses for the 203 downregulated genes specificially in GHMT/EP4 antagonist (ant) treatment are shown in (**d**).

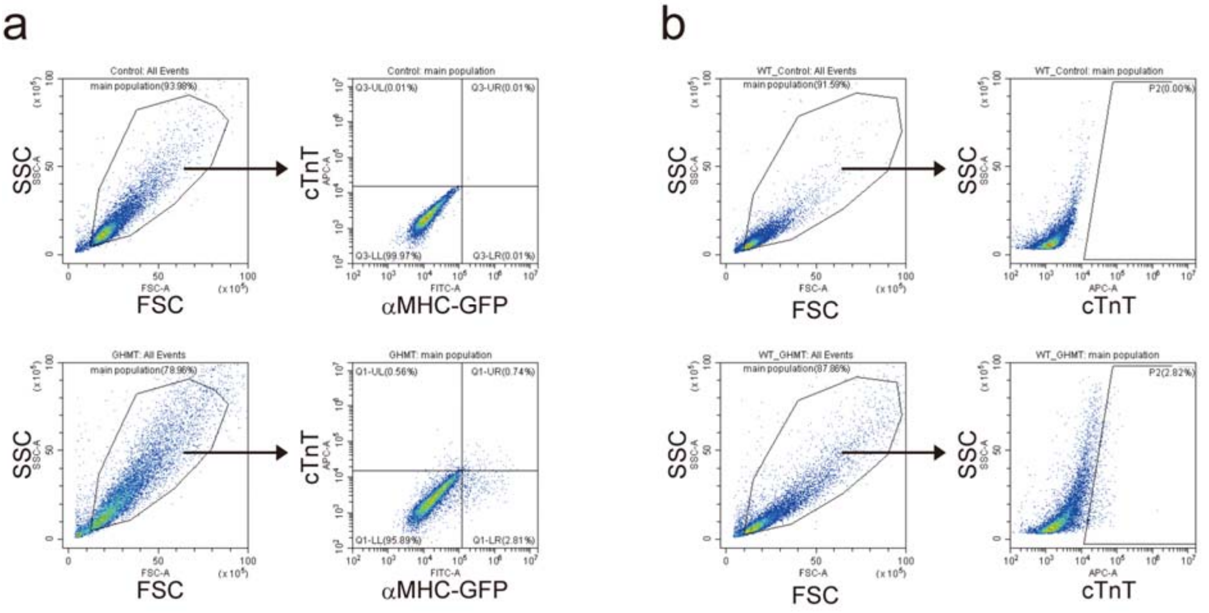

Supplementary Figure 6.

Representative Sequential Gating Strategies for Flow Cytometry Analysis.

(a) Gating strategy to determine αMHC-GFP^{+} and cTnT^{+} cells induced from αMHC-GFP fibroblasts, presented in Fig.1d, e, Fig.2a-c, Fig.3a-d, f-k, Fig.4a-e, Fig.5f, g, Fig.6h-j, Fig.7b, c, g-j, Supplementary Figure.1a, b, Supplementary Figure.2a, b, Supplementary Figure.3b, d-g and Supplementary Figure.4d, e.

(b) Gating strategy to determine cTnT^{+} cells induced from wild type or EP4-knockout fibroblasts, presented in Fig.4f, g.

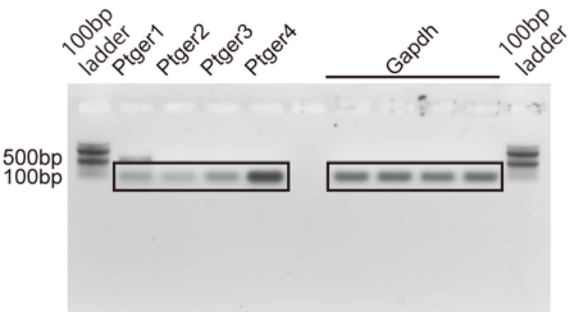

Supplementary Figure 7.

Uncropped scan image for Supplementary Figure 3a.

Supplementary Table 1. Related to Methods.

qRT-PCR primer sequences for SYBR green technology (TOYOBO).

Gene	Forward primer	Reverse primer
Ccl2	5′-AGGTCCCTGTCATGCTTCTG-3′	5′-CGTTAACTGCATCTGGCTGA-3′
Ccl8	5′-TCTACGCAGTGCTTCTTTGCC-3′	5′-AAGGGGGATCTTCAGCTTTAGT-3′
Gapdh	5′-AGCTTGTCATCAACGGGAAG-3′	5′-TTTGATGTTAGTGGGGTCTCG-3′

qRT-PCR primer sequences for Universal Probe Library (Roche).

Gene	Forward primer	Reverse primer	Probe no.
Actc1	5′-CCGATCGTATGCAAAAGGAA-3′	5′-CTGGAAGGTGGACAGAGAGG-3′	58
Ccr1	5′-GACACATTGAGCATCCAAGG-3′	5′-CAAAACAAAACTAACAGATTGGTCA-3′	51
Col12a1	5′-CCAGGTCCTCCTGGATATTG-3′	5′-AAATTTGTTAGCCGGAACCTG-3′	89
Cxcl11	5′-CTGTAGCCCACGTCGTAGC-3′	5′-TTGAGATCCATGCCGTTG-3′	25
Cxcl14	5′-TCTGCAAAGAGAGATCTCCAAAG-3′	5′-CGCCCCTGTTTGAACATAAG-3′	13
Cxcl3	5′-TGTGTGGACAAAATACTCTGGAA-3′	5′-GTGGGGTAGGCTTCTGTGAA-3′	58
Gapdh	5′-AGCTTGTCATCAACGGGAAG-3′	5′-TTTGATGTTAGTGGGGTCTCG-3′	9
Il1b	5′-ACCCTGCAGTGGTTCGAG-3′	5′-TTGCCATAGCTGCTTCAGAC-3′	55
Il6	5′-ACCCTGCAGTGGTTCGAG-3′	5′-TTGCCATAGCTGCTTCAGAC-3′	55
Il33	5′-GAAGCTGTGGGGAGATCG-3′	5′-TTTCAAGCACGCCTCTCTCT-3′	51
Myh6	5′-CGCATCAAGGAGCTCACC-3′	5′-CCTGCAGCCGCATTAAGT-3′	6
Nppa	5′-CACAGATCTGATGGATTTCAAGA-3′	5′-CCTCATCTTCTACCGGCATC-3′	25
Ryr2	5′-TTCAACACGCTCACGGAGTA-3′	5′-GTGCCAGGCTCTGCTGAT-3′	81
Snai1	5′-CTTGTGTCTGCACGACCTGT-3′	5′-CAGGAGAATGGCTTCTCACC-3′	81
Timp1	5′-GCAAAGAGCTTTCTCAAAGACC-3′	5′-AGGGATAGATAAACAGGGAAACACT-3′	76

TaqMan probes (Applied Biosystems).

**Target gene**	**Assay ID**
Gja1	Mm01179639_s1
Il1r1	Mm00434237_m1
Kcnd2	Mm01161732_m1
Pln	Mm00452263_m1
Ptger1	Mm00443098_g1
Ptger2	Mm00436051_m1
Ptger3	Mm01316856_m1
Ptger4	Mm00436053_m1
Ptgs1	Mm00477214_m1
Ptgs2	Mm00478374_m1
Scn5a	Mm00451971 m1
Slc8a1	Mm01232254_m1
Tnnc1	Mm00437115_g1

Supplementary Table 2. Related to Fig. 1c.

List of Compounds promoting Cardiac Reprogramming in Postnatal TTFs.

**ID number**	**Formula**	**Structure**	**Source**
QA-6161	C14H10Cl2NNaO2	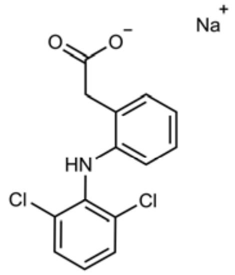	Combi-Blocks
STK081095	C16H11N3O2S2	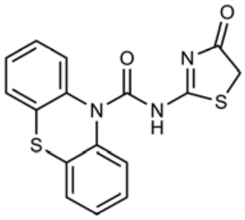	Vista-M Labolatory
STK833231	C17H16N2O	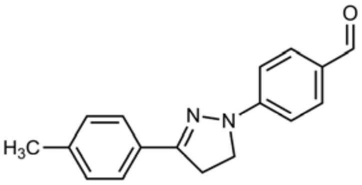	Vista-M Labolatory
STK825882	C17H18N2O5S	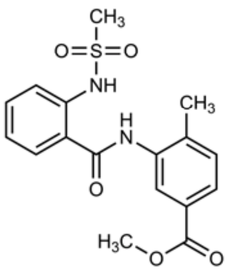	Vista-M Labolatory
