# Supplementary material for: Role of cyclooxygenase-2-mediated prostaglandin E2-prostaglandin E receptor 4 signaling in cardiac reprogramming
Source: Nat Commun. 2019 Feb 20;10:674. doi: 10.1038/s41467-019-08626-y (PMC6382796; doi:10.1038/s41467-019-08626-y)
Supplement: Supplementary file 3 — Description of Additional Supplementary Files [file 41467_2019_8626_MOESM3_ESM.pdf]

Description of Additional Supplementary Files

Supplementary Movie 1.
Spontaneous Ca^{2+} oscillations in GHMT/diclofenac-induced iCMs cultured for 6 weeks.

Supplementary Movie 2.
Spontaneous beating of GHMT/diclofenac-induced iCMs cultured for 6 weeks.
